# Predicting the immune landscape of invasive breast carcinoma based on the novel signature of immune‐related lncRNA

**DOI:** 10.1002/cam4.4189

**Published:** 2021-08-11

**Authors:** Shuang Shen, Xin Chen, Xiaochi Hu, Jinlong Huo, Libo Luo, Xuezhi Zhou

**Affiliations:** ^1^ Department of Breast & Thyroid Surgery Third Affiliated Hospital of Zunyi Medical University/First People’s Hospital of Zunyi Zunyi Guizhou China

**Keywords:** breast cancer, immunotherapy, LncRNA, risk score, TCGA, tumor‐infiltrating immune

## Abstract

**Background:**

The composition of the population of immune‐related long non‐coding ribonucleic acid (irlncRNA) generates a signature, irrespective of expression level, with potential value in predicting the survival status of patients with invasive breast carcinoma.

**Methods:**

The current study uses univariate analysis to identify differentially expressed irlncRNA (DEirlncRNA) pairs from RNA‐Seq data from The Cancer Genome Atlas (TCGA). 36 pairs of DEirlncRNA pairs were identified. Using various algorithms to construct a model, we have compared the area under the curve and calculated the 5‐year curve of Akaike information criterion (AIC) values, which allows determination of the threshold indicating the maximum value for differentiation. Through cut‐off point to establish the optimal model for distinguishing high‐risk or low‐risk groups among breast cancer patients. We assigned individual patients with invasive breast cancer to either high risk or low risk groups depending on the cut‐off point, re‐evaluated the tumor immune cell infiltration, the effectiveness of chemotherapy, immunosuppressive biomarkers, and immunotherapy.

**Results:**

After re‐assessing patients according to the threshold, we demonstrated an effective means of distinguish the severity of the disease, and identified patients with different clinicopathological characteristics, specific tumor immune infiltration states, high sensitivity to chemotherapy,wellpredicted response to immunotherapy and thus a more favorable survival outcome.

**Conclusions:**

The current study presents novel findings regarding the use of irlncRNA without the need to predict precise expression levels in the prognosis of breast cancer patients and to indicate their suitability for anti‐tumor immunotherapy.

## Graphical abstract

The manuscript is an immune‐related lncRNA pairing strategy to construct a risk assessment model. We did not use precise expression levels of the target lncRNAs in the signature.

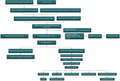

## 1 INTRODUCTION

Breast cancer is the most common type of cancer and the most likely to cause death in women.^1^ In 2020, new breast cancer cases worldwide numbered 2.3 million, surpassing lung cancer, and constituting the most frequent cancer type globally. Moreover, breast cancer accounts for 685,000 deaths annually throughout the world and has the fifth highest mortality rate of any cancer type.^2^

There are many risk factors that contribute to breast cancer, such as high levels of female sex hormones, history of breast cancer among first‐degree relatives, and germline mutations in the BRCA gene.^3^ Recent advances in the development of techniques in molecular biology and genomics have enabled the study of the causes and treatments of breast cancer at the genetic level. The discovery of immune checkpoints has exposed new targets for the treatment of malignant tumors, such as immune checkpoint inhibition (ICI) therapy for programmed cell death protein 1 (PD‐1) and its ligand PD‐L1.^4^, ^5^ Such therapy has been used for solid tumors of various origins and has significantly improved patient survival.^6^

Atezolizumab plus nab‐paclitaxel regimen was approved by the FDA in 2019 as the first‐line treatment for advanced triple‐negative breast cancer (TNBC). Overall survival (OS) and progression‐free survival (PFS) rates in patients treated with atezolizumab plus nab‐paclitaxel in phase III IMpassion130 trial were significantly improved.^7^Moreover, the KEYNOTE‐173 clinical trial demonstrated that high expression of PD‐L1 in tumor cells had a positive correlation with overall remission rates,^8^ including complete remission, in TNBC patients who received immunotherapy‐based neoadjuvant treatment. Therefore, there is great potential for the evaluation of TILs (tumor‐infiltrating lymphocytes) to enable screening for patients who will benefit from immunotherapy.

LncRNAs account for a considerable proportion of the human transcriptome. They regulate a number of physiological and pathological processes by interacting with DNA, mRNA, ncRNAs, and proteins. Many previous studies have shown that lncRNAs regulate numerous biological processes concerned with the occurrence and development of tumors.^9^, ^10^ For example, abnormal expression of lncRNA leads to significant changes in gene expression during the malignant transformation of breast tissue.^11^ Cai et al.^12^ also confirmed through in vivo and in vitro experiments that lncRNA CCAT2 can regulate the Wnt signaling pathway, which in turn leads to a progressive proliferation of breast cancer cells. Furthermore, the content of lncRNA in breast cancer metastases is often significantly altered compared with non‐cancerous tissues and with primary tumors and its expression level is often correlated with invasiveness and prognosis.^13^

Recent evidence has shown that lncRNA can not only change gene expression on a transcriptome level, but may also affect the immune microenvironment of the tumor.^14^, ^15^, ^16^ Such changes contribute to the cancer phenotype when lncRNA regulates gene expression related to immune cell activation, allowing the infiltration of tumor cells. Since lncRNAs influence tumor occurrence and development, a study of the relevant mechanisms is expected to have better predictive and prognostic value for tumor evaluation and treatment. Wang et al.^17^ identified nine immune‐related lncRNAs (irlncRNA) allowing the development of a standard for the assessment of the prognosis of patients with anaplastic glioma. Zhou et al.^18^established a novel lncRNA‐focused expression profile (LFES) comprising 11 survival‐related lncRNAs to help tailor treatment and improve the prognosis of endometrial cancer patients. In addition, Fang et al.^19^ analyzed the mRNA, MicroRNA (miRNA), and lncRNA of patients with head and neck squamous cell carcinoma, combined with clinical data, establish immune‐related standards to predict the survival rate of patients with this tumor. By the analysis of tumor and paracancerous tissues, Weng et al.^20^ study on patients with esophageal squamous cell carcinoma (ESCC) found that seven lncRNAs can be used as potential prognostic biomarkers, the use of which along with the TNM staging system may help treatment decisions.

The combination of two biomarkers shows improved accuracy over single gene markers when considering models of cancer diagnosis,^21^ although past models have often overlooked the impact of multiple biomarker effects. The current study employs the novel modeling algorithm developed by Hong et al.^22^ to construct, through pairing and iteration, irlncRNA signatures without the requirement for any specific expression level. We evaluated the predictive value and diagnostic efficacy for chemotherapy efficacy, tumor immune infiltration, and immunotherapy response in patients with invasive breast carcinoma.

## 2 MATERIALS AND METHODS

### 2.1 Acquisition, preparation, and differential expression analysis of transcriptome data

All data analysis was performed by R software (version 4.0.3). FPKM (fragments per kilobase million) data were obtained from the TCGA BRCA project (https://tcga‐data.nci.nih.gov/tcga/) and used to construct the transcriptome. The GTF file obtained through Ensembl (http://asia.ensembl.org) was used to distinguish lncRNA from mRNA. Then obtain of irlncRNA through the correlation of the certified immune‐related genes (ir‐genes) (Table S1) in the ImmPort database (http://www.immport.org).^23^ The correlation between all lncRNAs and ir‐genes was analyzed by co‐expression strategy, and the correlation coefficient condition for identifying irlncRNA was set to corFilter was more than 0.4, pvalueFilter was less than 0.001. Identification of DEirlncRNA: We used the R package *limma* to perform differential analysis of the expression of irlncRNA in tumor and norm patients. Set the screening threshold to false discovery rate (fdrFilter) <0.05 and log fold change (fcFilter) > 1.0.

### 2.2 Definition of Pairing of DEirlncRNAs

Construct a 0 or 1 matrix using the DEirlncRNAs from the previous stage. The strategy for constructing DEirlncRNAs pairs is as follows: The DEirlncRNAs were cyclically singly paired (iteration loop), assuming Z = DEirlncRNA X + DEirlncRNA Y, if the expression level of DEirlncRNA X is higher than DEirlncRNA Y, Z is defined as 1, otherwise Z is defined as 0. Then, the constructed 0‐or‐1 matrix was further screened. Set screening conditions: When quantities of DEirlncRNA pairs represent more than 20% of the total number of pairs, an effective match had been achieved, because the pair without a certain rank cannot properly predict the patient's survival outcome. The final screened matrix (Table S2) was used for the next step.

### 2.3 Selection of patients’ clinical data

Obtain clinical data of patients with invasive breast cancer through TCGA's BRCA project (Table 1). One thousand and thirty‐four patients with complete follow‐up information and survival time ≥30 days performed a survival analysis, 705 patients with complete clinicopathological data performed clinical correlation analysis.

**TABLE 1:** Clinical characteristics of patients with BC in TCGA

Characteristic	Levels	Overall
*n*		1083
T stage, *n* (%)	T1	277 (25.6)
	T2	629 (58.2)
	T3	139 (12.9)
	T4	35 (3.2)
N stage, *n* (%)	N0	514 (48.3)
	N1	358 (33.6)
	N2	116 (10.9)
	N3	76 (7.1)
M stage, *n* (%)	M0	902 (97.8)
	M1	20 (2.2)
Pathologic stage, *n* (%)	Stage I	181 (17.1)
	Stage II	619 (58.4)
	Stage III	242 (22.8)
	Stage IV	18 (1.7)
Age, *n* (%)	≤60	601 (55.5)
	>60	482 (44.5)
PR status, *n* (%)	Negative	342 (33.1)
	Indeterminate	4 (0.4)
	Positive	688 (66.5)
ER status, *n* (%)	Negative	240 (23.2)
	Indeterminate	2 (0.2)
	Positive	793 (76.6)
HER2 status, *n* (%)	Negative	558 (76.8)
	Indeterminate	12 (1.7)
	Positive	157 (21.6)
PAM50, *n* (%)	Normal	40 (3.7)
	LumA	562 (51.9)
	LumB	204 (18.8)
	Her2	82 (7.6)
	Basal	195 (18)
OS event, *n* (%)	Alive	931 (86)
	Dead	152 (14)
Histological type, *n* (%)	Infiltrating Ductal Carcinoma	772 (79)
	Infiltrating Lobular Carcinoma	205 (21)
Age, median (IQR)		58 (48.5, 67)

### 2.4 Establishing a risk model for evaluating riskScore

Combine the previously acquired DEirlncRNAs pairs matrix with the patient's clinical information. Single‐factor analysis was performed to screen out the DEirlncRNAs pairs related to the patient's prognosis (the filter condition was set to *p* < 0.01). Then, 10‐fold cross‐validated Lasso regression was performed, with a *p* value of 0.05. Lasso regression was performed 1000 cycles and each cycle was randomly stimulated 1000 times. Next, record the frequency of each pair in the Lasso regression model with 1000 repetitions, and select the pairs with a frequency of more than 100 times to perform the Cox proportional hazard regression analysis and build the model (Table S3). Calculate the AUC value of each model and draw the curve. When the curve reaches the highest point, that is, when the AUC value is the largest, the calculation procedure is terminated and the model is the best candidate. ROC curves were constructed for 1, 3, 5, and 7 years. Used the formula ($\text{RiskScore}=\sum_{i=1}^{k}\beta iSi$) to calculate riskscore for all clinical cases. By evaluating the AIC value of each point in the 5‐year ROC, the maximum inflection point could be identified and was considered to indicate the threshold for the distinction between high or low risk. We calculated risk scores for all acceptable patients and used the threshold to re‐differentiate the high‐risk and low‐risk groups in the cohort.

### 2.5 Validation of the risk model

To verify the model, we used Kaplan–Meier analysis to validate differences in survival between the high‐risk group and the low‐risk group, and to visualize the survival curve. The R packages we used include *survival*, *survivor*, *glmnet*, *survminer*, *pHeatmap*, and *pbapply*.

We used a chi‐square test to analyze the relationship between the model and different clinicopathological characteristics to verify the clinical value of the model. Visualized with a bar chart and mark as low points: <0.001 = ***, <0.01 = **, and <0.05 = *. The Wilcoxon rank‐sum test was used to calculate the difference in riskScore between different groups displaying the different clinicopathological characteristics. The block diagram shows the results of the analysis.

To confirm whether the model has utility as an independent clinical prognostic predictor, the riskScore and clinicopathological characteristics were compared by univariate and multivariate Cox regression analysis. The results are shown by means of Forest plots. In addition, we constructed a nomogram integrating prognostic signature to predict the 3, 5, and 7‐years OS of BC patients The R packages we used include *ggupbr*, *survival*, *pHeatmap*, *and regplot*.

### 2.6 Study on tumor‐infiltrating immune cells

To compare the characteristics of immune cell infiltration between high and low‐risk groups, we applied currently available software (including TIMER,^24^, ^25^CIBERSORT,^26^, ^27^ XCELL,^28^, ^29^ QUANTISEQ,^30^, ^31^ MCPcounter,^32^EPIC,^33^ and CIBERSORT‐ABS^34^) to the TCGA‐BRCA database. The Wilcoxon rank‐sum test was used to analyze the difference in the content of immune infiltrating cells between the high‐risk and low‐risk groups. Spearman correlation analysis was used to analyze the relationship between the immune infifiltrated cells and the riskScore values. The significance threshold was set to *p* < 0.05. The above results were visualized using the R *ggplot2* software package.

### 2.7 Clinical significance of the risk model for the treatment of invasive breast cancer

To evaluate the model in the clinical treatment of breast cancer, we calculated the IC50 for the chemotherapeutic drugs commonly used in the TCGA‐BRCA database. The NCCN (National Comprehensive Cancer Network) guidelines recommend the use of anti‐tumor drugs, such as paclitaxel, docetaxel, doxorubicin, cisplatin, Vinorelbine, Gemcitabine, Lapatinib, and Palbociclib for breast cancer treatment. The difference in IC50 between high‐risk and low‐risk groups was compared by the Wilcoxon signed‐rank test and R's *pRRophetic* and *ggplot2* were used.

### 2.8 ICI‐related molecule expression and immunotherapy analysis

To quantify the relationship between our model and the expression level of ICI‐related genes, we performed *ggstatsplot* packaging and violin performance Plot visualization. To explore the relationship between riskScore and immunotherapy. We download immunotherapy antigen information from the Cancer Immunity Atlas (https://tcia.at/home). We used the immunotherapy antigen to calculate the four types (CTLA4_negative+PD‐1_negative,CTLA4_positive+PD‐1_positive, CTLA4_negative+ PD‐1_positive, CTLA4_positive+ PD‐1_negative) of immunophenoscore (IPS) with the BC patients.^35^

## 3 RESULTS

### 3.1 Identification of differentially expressed irlncRNA (DEirlncRNA)

**FIGURE 1:**
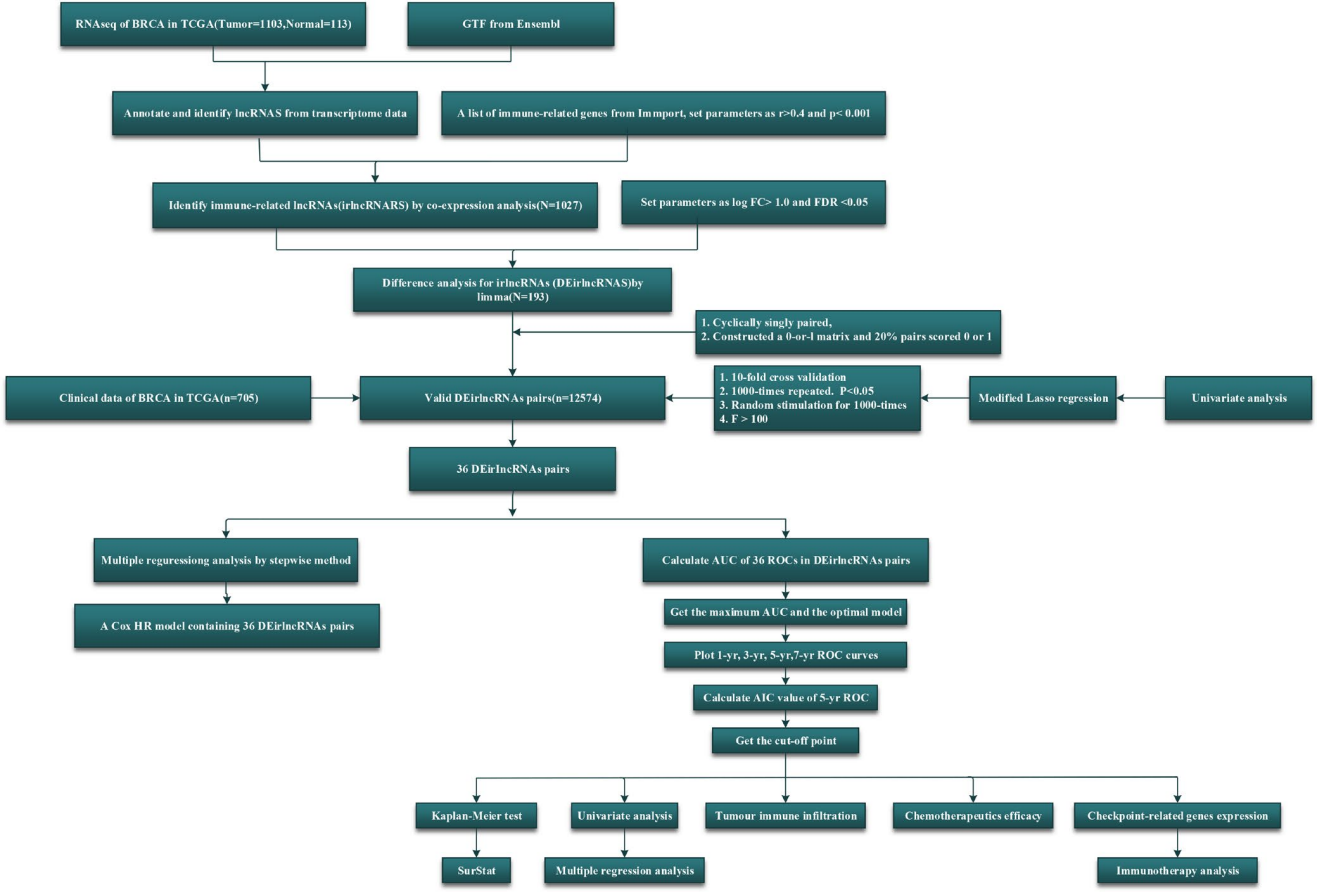
Flow chart of this study

**FIGURE 2:**
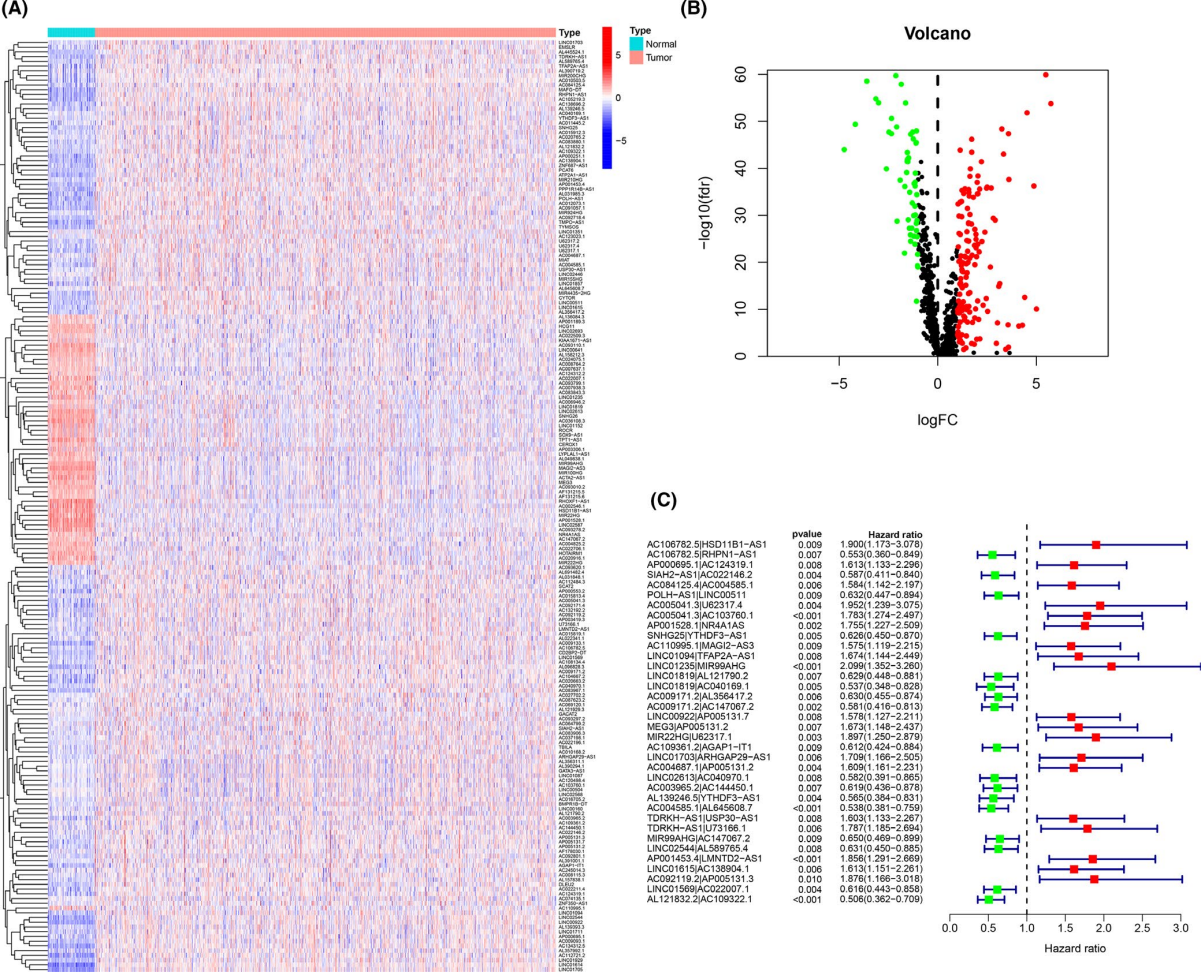
Identification of differentially expressed immune‐related lncRNAs (DEirlncRNAs) using TCGA datasets and annotation by Ensembl. (A and B) The heatmap (A) and volcano plot (B) are shown. (C) A forest map showed 36 DEirlncRNA pairs identified by Cox proportional hazard regression in the stepwise method

The study methods are summarized in Figure 1. We obtained data relating to 113 normal and 1103 invasive breast carcinoma samples from the TCGA‐BRCA database. A total of 819 irlncRNAs were identified (Table S4) of which 193 were classified as DEirlncRNAs (Figure 2A) with 54 being down‐regulated and 143 up‐regulated (Figure 2B).

### 3.2 Identification of DEirlncRNA pairs and establishment of risk assessment model

**FIGURE 3:**
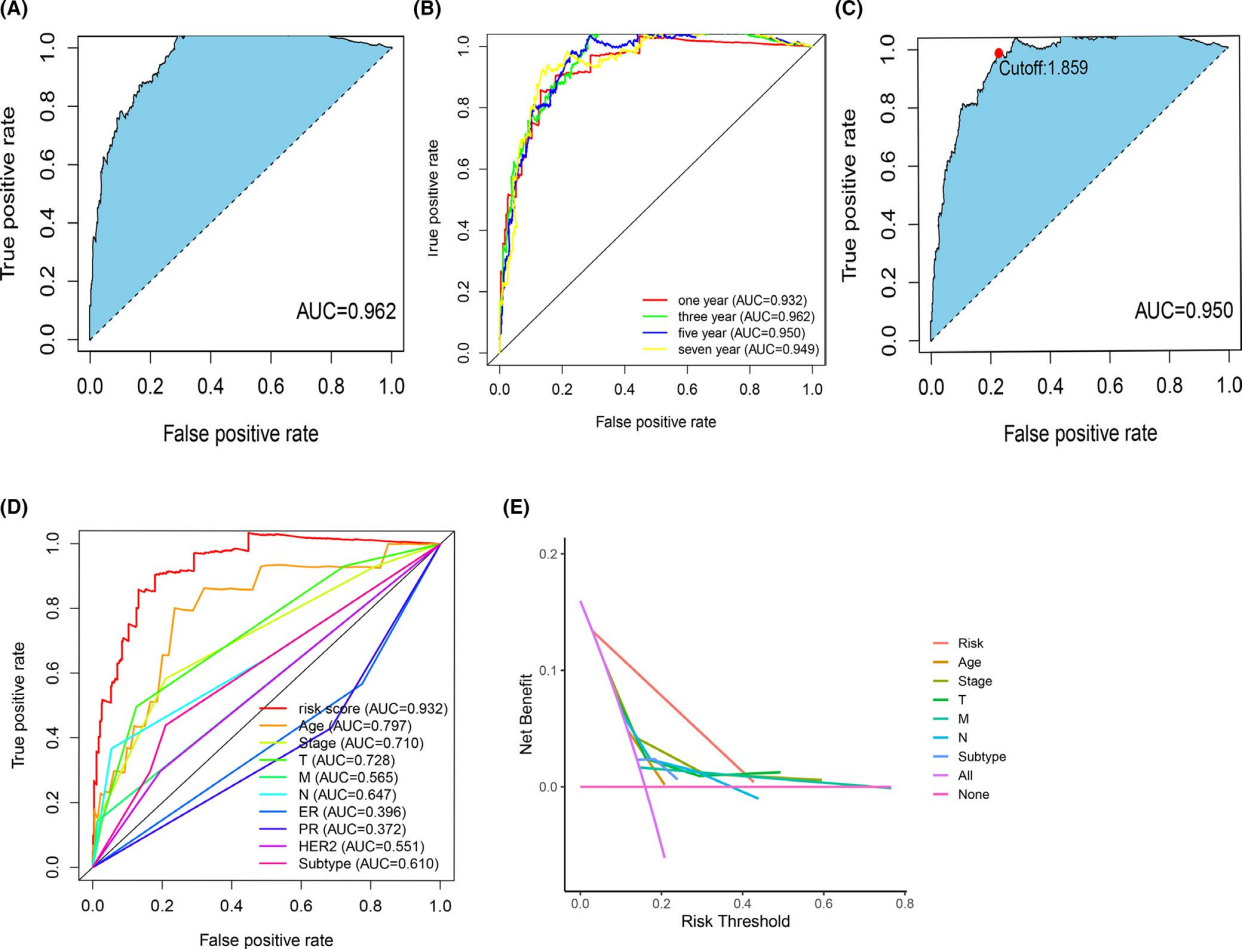
Establishment of a risk model to evaluate the RiskScore. (A) Plot a curve of every AUC value generated by ROCs of 36 DEirlncRNA pair models and identify the highest point of the AUC. The ROC of the optimal DEirlncRNA pair models was related to the maximum AUC. (B) The 1‐, 3‐, 5‐, and 7‐year ROC of the optimal model suggested that all AUC values were over 0.90. (C) RiskScore for 705 patients with invasive breast carcinoma; the maximum inflection point is the cut‐off point obtained by the AIC. (D) A comparison of 5‐year ROC curves with other common clinical characteristics showed the superiority of the riskScore. (E) The DCA (Decision Curve Analysis) of the risk factors

193 DEirlncRNAs through iterative loop, single factor test, improved Lasso regression analysis and Cox proportional hazard regression analysis, we constructed a risk model composed of 36 pairs of DEirlncRNA (Figure 2C). We calculated the area under the curve (AUC) for each receiver operating characteristic (ROC) curve of 36 pairs and a plot of the AUC data produced the value of the highest point as 0.962 (Figure 3A). All AUC values exceeded 0.9 by 1, 3, 5, and 7‐year ROC curves (Figure 3B). We also used the Akaike Information Criterion (AIC) value to identify the maximum inflection point as the critical point of the 5‐year ROC curve (Figure 3C). Compared to the 5‐year ROC curve with other clinical characteristics, the risk model showed more favorable performance (Figure 3D,E).

### 3.3 Clinical evaluation by the risk assessment model

**FIGURE 4:**
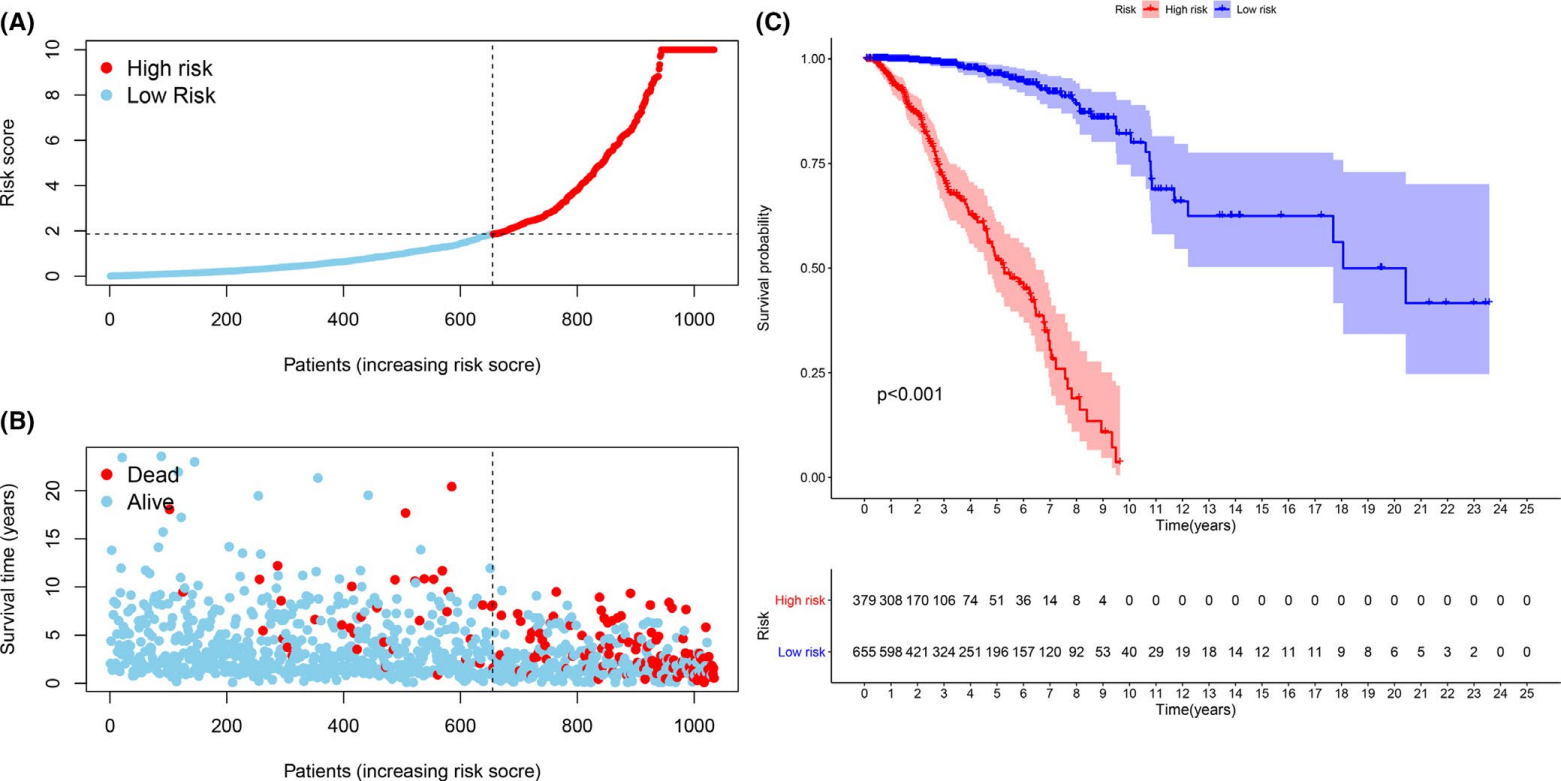
Validation of the risk model. (A and B) Risk scores (A) and survival outcome (B) of each case are shown. (C) Patients in the low‐risk group experienced a longer survival time tested by the Kaplan–Meier test

**FIGURE 5:**
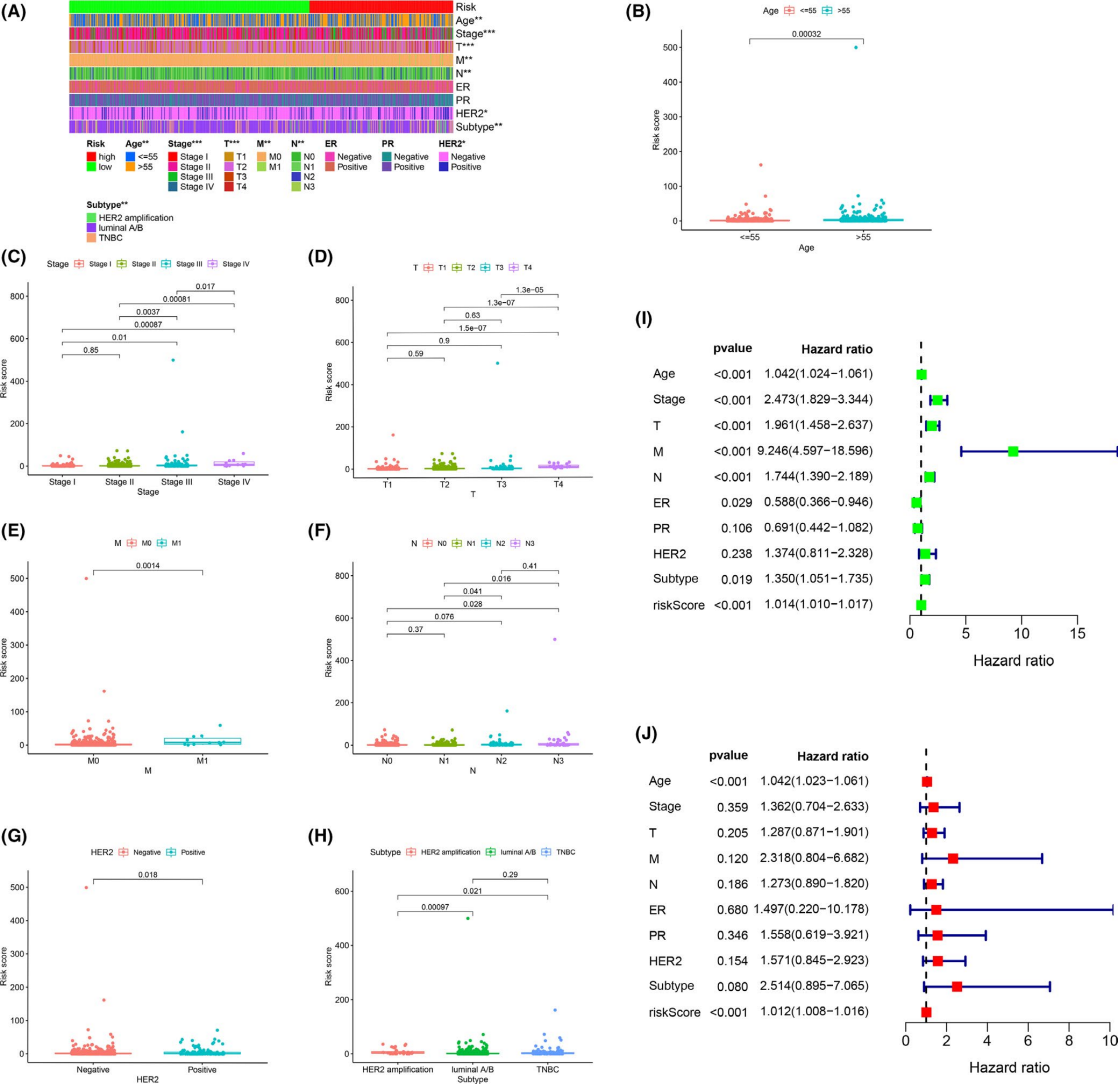
Clinical Evaluation by the risk assessment model. (A–H) A strip chart (A) along with the scatter diagram showed that (B) age, (C) clinical stage, (D) T stage, (E) M stage, (F) N stage, (G) Her2 status, and (H) subtype were significantly associated with the riskScore. (I) A univariate Cox hazard ratio analysis demonstrated that age (*p* < 0.001, HR =* *1.042, 95% CI [1.024–1.061]), clinical‐stage (*p* < 0.001, HR =* *2.473, 95% CI [1.829–3.344]), T stage (*p* < 0.001, HR =* *1.961, 95% CI [1.458–2.637]), M stage (*p* < 0.001, HR =* *9.246, 95% CI [4.597–18.596]), N stage (*p* < 0.001, HR =* *1.744, 95%CI [1.390−2.189]), and riskScore (*p* < 0.001, HR =* *1.014, 95%CI [1.010−1.017]).(J) Only Only age (*p* < 0.001, HR =* *1.042, 95% CI [1.024–1.061]) and RiskScore (*p* < 0.001, HR =* *1.012, 95% CI [1.008–1.016]) presented as an independent prognostic predictor by multivariate Cox regression

**FIGURE 6:**
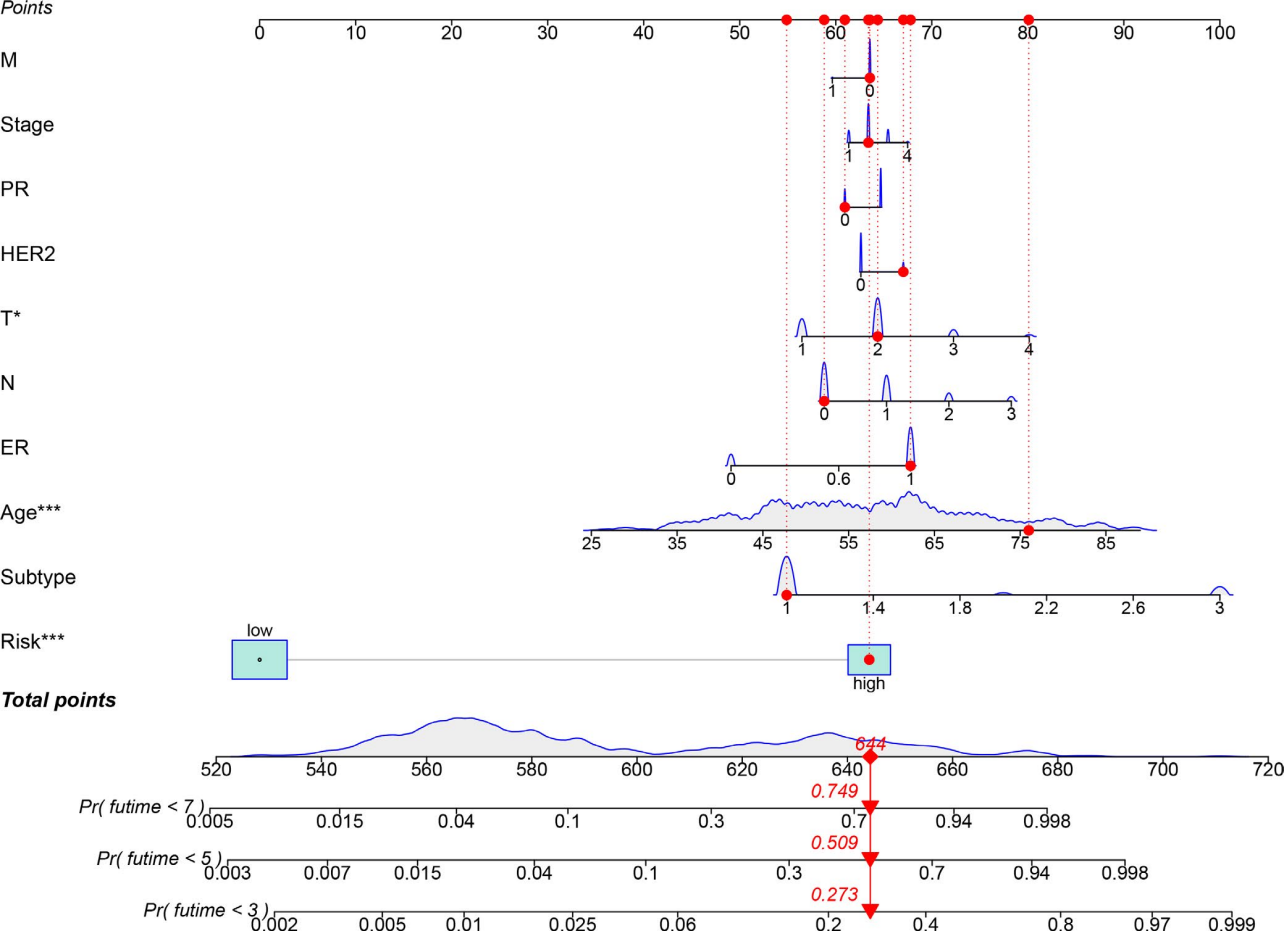
A nomogram integrating prognostic signature to predict the 3, 5, and 7‐years OS of BC patients

According to our threshold, 379 cases were assigned to the high‐risk group and 655 cases to the low‐risk group. The risk score and survival rate of each case are shown in Figure 4A,B, respectively. The values indicate that clinical outcomes of patients in the low‐risk group are better than those in the high‐risk group. The Kaplan–Meier analysis in Figure 4C shows that patients in the low‐risk group survived longer than those in the high‐risk group (*p* < 0.001). The strip chart (Figure 5A) and the accompanying scatter plot show that age (Figure 5B), clinical stage (Figure 5C), T stage (Figure 5D), M stage (Figure 5E), N stage (Figure 5F), Her2 status (Figure 5G), and subtype (Figure 5H) are significantly related to risk. Next, we demonstrated age (*p* < 0.001, HR = 1.042, 95% CI [1.024–1.061]), clinical‐stage (*p* < 0.001, HR = 2.473, 95% CI [1.829–3.344]), T stage (*p* < 0.001, HR = 1.961, 95% CI [1.458–2.637]), M stage (*p* < 0.001, HR = 9.246, 95% CI [4.597–18.596]), N stage (*p* < 0.001, HR = 1.744, 95%CI [1.390−2.189]), and riskScore (*p* < 0.001, HR = 1.014, 95%CI [1.010−1.017]) showed statistical differences (Figure 5I). Only age (*p* < 0.001, HR = 1.042, 95% CI [1.024–1.061]) and riskScore (*p* < 0.001, HR = 1.012, 95% CI [1.008–1.016]) were confirmed as independent prognostic indicators (Figure 5J). Table S5 shows the detailed values of univariate and multivariate Cox regression analysis. The hybrid nomogram (Figure 6) that combines clinicopathological characteristics and risk model was stable and accurate, thus may be applied in clinical practice for BC patients.

### 3.4 Association of risk model with tumor‐infiltrating immune cells

**FIGURE 7:**
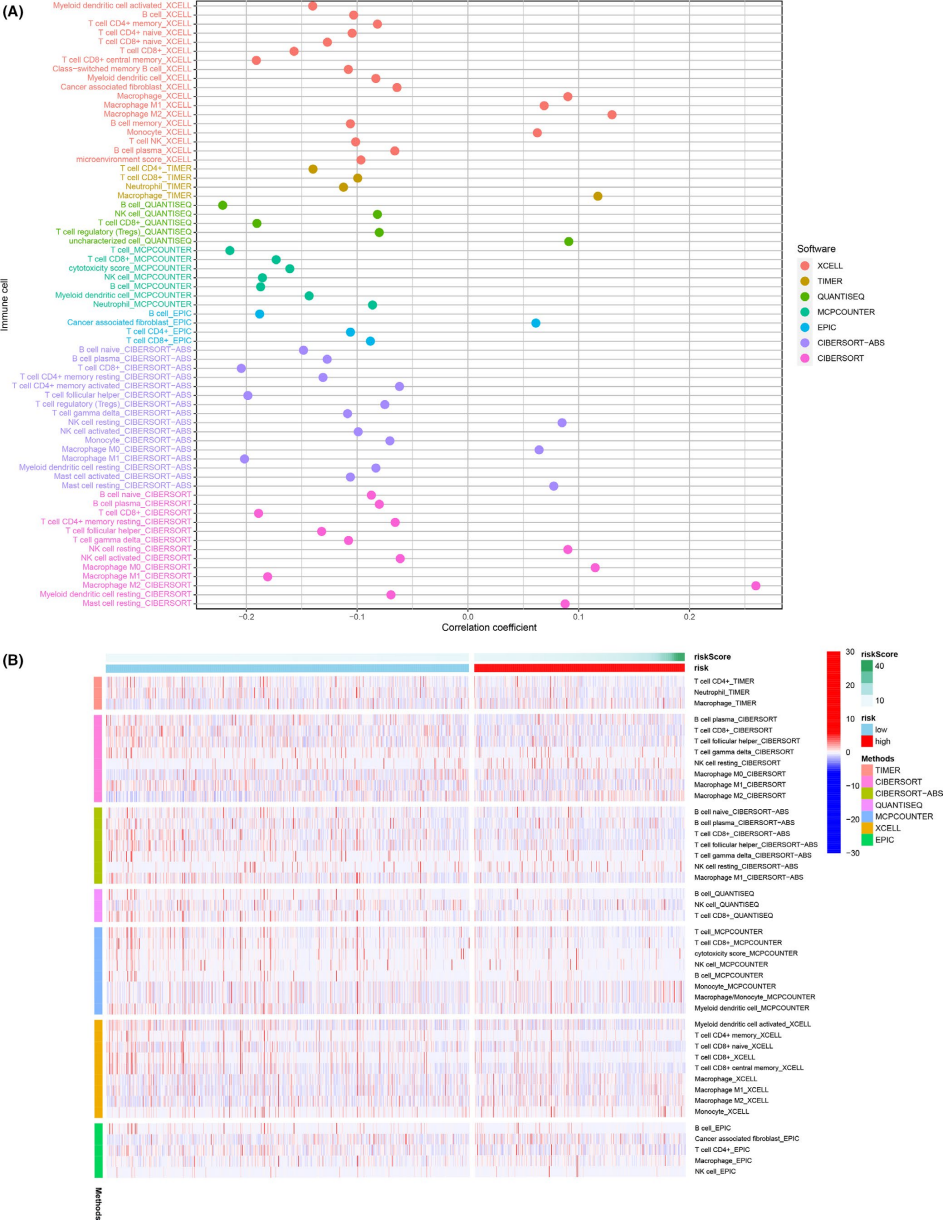
Estimation of tumor‐infiltrating cells by the risk assessment model. (A) In higher negative correlation with mast cells, macrophages, T cells, and fibroblasts in the high‐risk group and a higher negative correlation with tumor‐infiltrating immune cells, such as B cells, CD4+ T cells, and CD8+ T cells in the low‐risk group, as shown by Spearman correlation analysis. (B) Heatmap for immune responses based on CIBERSORT, ESTIMATE, MCPcounter, ssGSEA, and TIMER algorithms among high and low‐risk groups

Since lncRNA shows considerable overlap with immune‐related genes, we investigated whether our lncRNA model might be related to the tumor immune microenvironment. We found that positively associated with Macrophage, Macrophage M0, Macrophage M2, and Monocyte in the high‐risk group (Figure S1), a higher positively associated with tumor‐infiltrating immune cells, such as B cells, Macrophage M1, CD4+ T cells and CD8+ T cells in the low‐risk group (Figure S2). Figure 7A,B show the correlation of tumor‐infiltrating immune cells. The results are listed in Table S6.

### 3.5 Correlation analysis between risk model and chemotherapy

**FIGURE 8:**
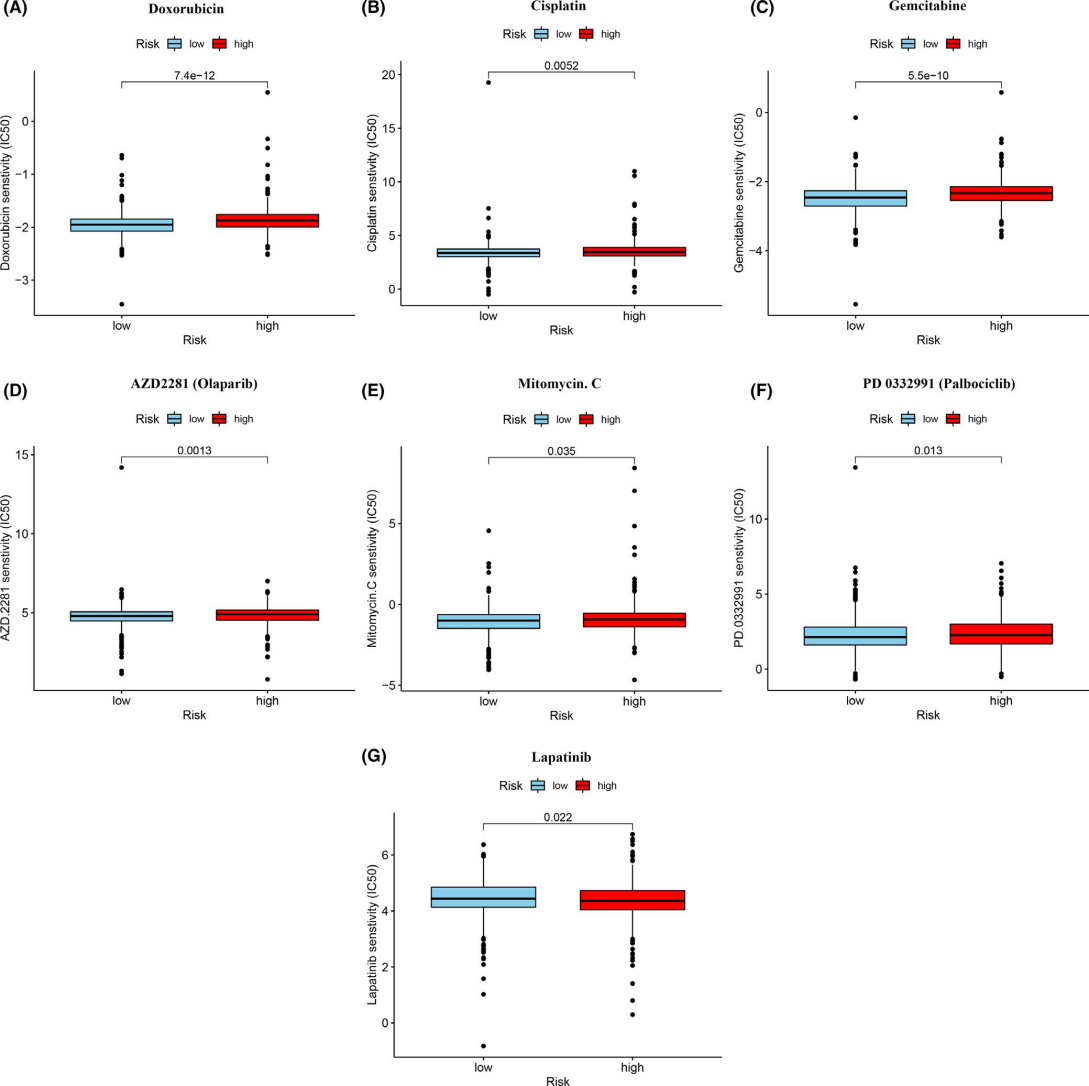
Correlation analysis between risk model and chemotherapy. The model acted as a potential predictor for chemosensitivity as low‐risk scores were related to a lower IC50 for chemotherapeutics such as doxorubicin, cisplatin, gemcitabine, AZD2281 (Olaparib), Mitomycin. C, and PD 0332991 (Palbociclib), whereas they were related to a higher IC50 for lapatinib

We explored the relationship between risk and efficacy of common chemotherapy in the TCGA‐BRCA database We found that high‐risk group is related to the higher IC50 of chemotherapy drugs, such as doxorubicin (*p* < 0.001), cisplatin (*p* < 0.01), gemcitabine (*p* < 0.001), AZD2281 (Olaparib)) (*p* < 0.01), Mitomycin. C (*p* < 0.05), and PD 0332991 (Palbociclib) (*p* < 0.05). The IC50 of Lapatinib was found to be lower (*p* < 0.05). These findings indicated that the model can be used as a potential predictor of chemotherapeutic sensitivity (Figure 8).

### 3.6 Association of risk model between immunosuppressive molecules and immunotherapy

**FIGURE 9:**
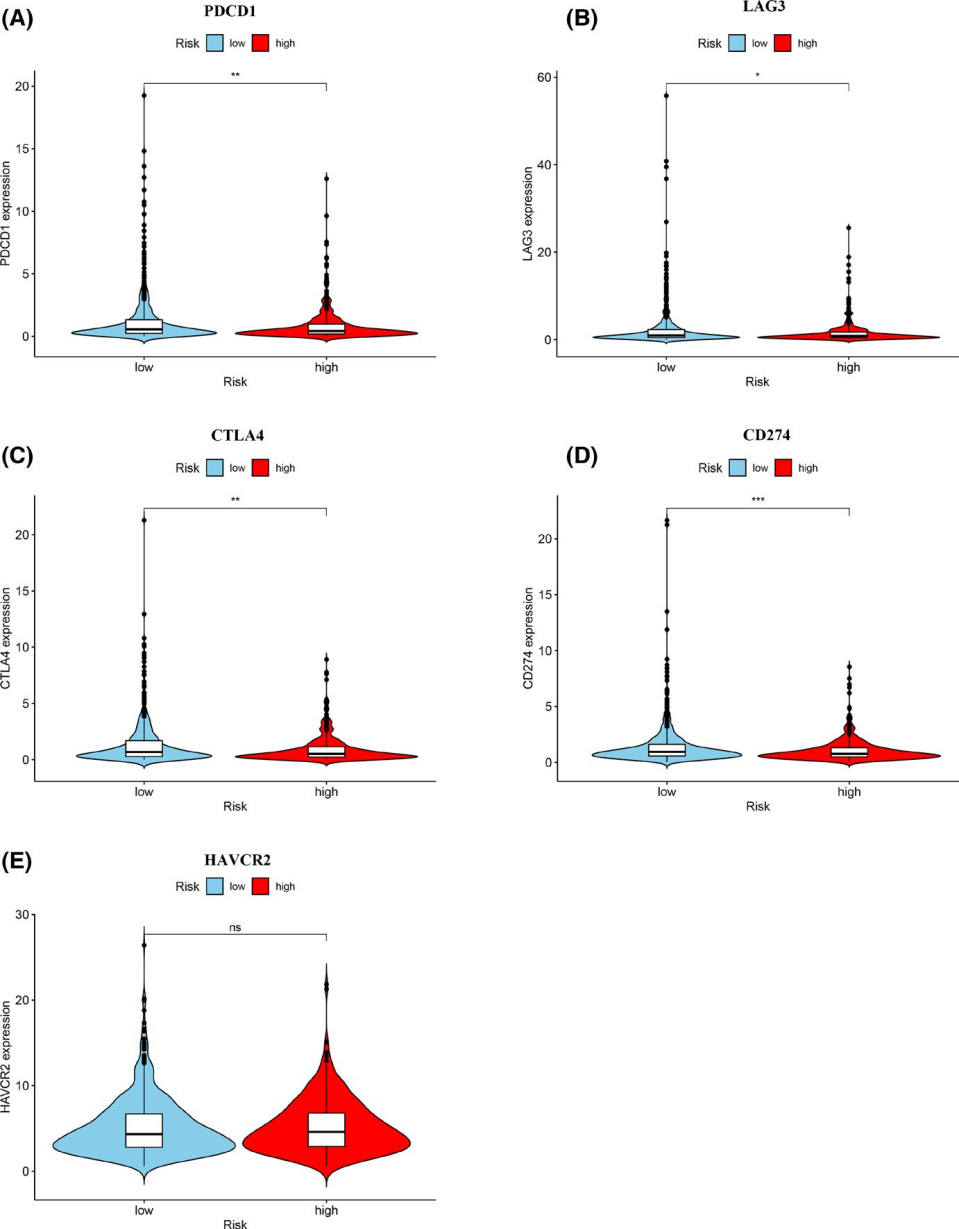
Immunosuppressed molecules by the risk assessment model. (A–E) Low‐risk group was positively correlated with upregulated (A) PDCD1, (B) LAG3, (C) CTLA4, (D) CD274, and (E) HAVCR2 levels, whereas the latter one showed no statistical difference in patients with invasive breast carcinoma

**FIGURE 10:**
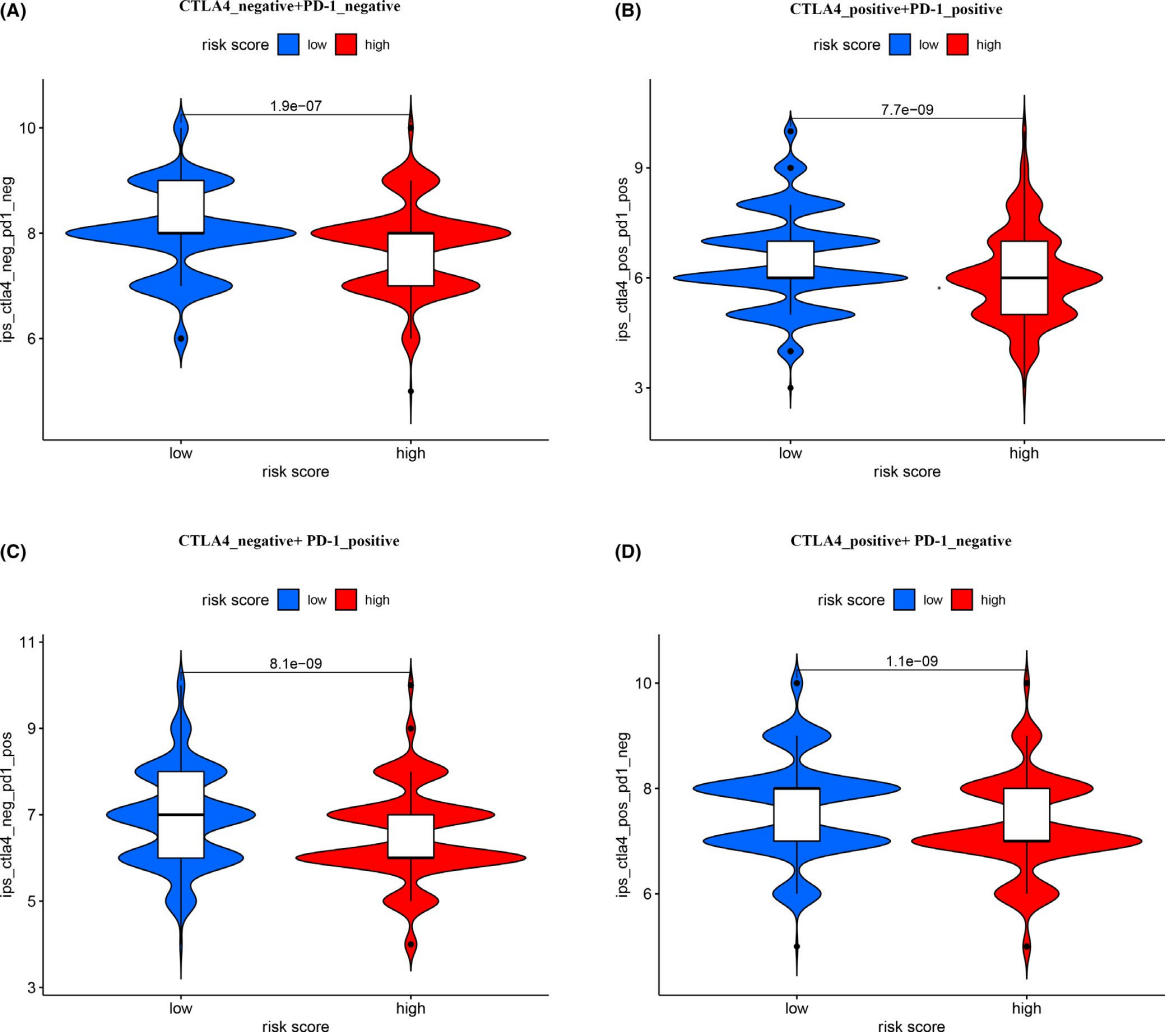
Association of risk model with Immunotherapy. IPS comparison between low‐risk groups and high‐risk groups in BC patients in the CTLA4 negative/positive or PD‐1 negative/positive groups. CTLA4_positive or PD1_positive represented anti‐CTLA4 or anti‐PD‐1/PD‐L1 therapy, respectively

ICI is used in clinical practice to treat invasive breast carcinoma and the results of our investigation into whether our risk model can be related to ICI‐related biomarkers. We found that low‐risk group was positively correlated with high expression of PDCD1 (*p* < 0.01, Figure 9A), LAG3 (*p* < 0.05, Figure 9B), CTLA4 (*p* < 0.01, Figure 9C), and CD274 (*p* < 0.001, Figure 9D), but no statistical differences in HAVCR2 (*p* > 0.05, Figure 9E). In addition, among the four types of immunophenoscore, we found that the IPS in the low‐risk group was significantly higher in the high‐risk group (both *p* < 0.0001) (Figure 10). These results indicated that patients from low‐risk group showed a higher positive response to immunotherapy.

## 4 DISCUSSION

Current research has largely focused on predicting or evaluating the prognosis of patients with malignant tumors by quantifying the expression levels of various coding and non‐coding RNAs based on the analysis of transcripts.^36^, ^37^ In this study, we used an immune‐related lncRNA pairing strategy to construct a risk assessment model instead of using precise expression levels of the target lncRNAs. To the best of our knowledge, this is the first study to employ the strategy of comparing pairs of lncRNAs to create a risk assessment model for breast cancer.

We obtained lncRNA data from TCGA and used differential co‐expression analysis to identify DEirlncRNA pairs. We employed a series of modifying algorithms to screen for DEirlncRNA pairs. We calculated the AUC value for each ROC and the AIC value of each point on the AUC to determine the optimal threshold to distinguish between high‐risk and low‐risk breast cancer patients. We also validated our model by comparison with a variety of clinical benchmarks, including survival, clinicopathological characteristics, molecular typing of breast cancer, tumor‐infiltrating immune cells, chemotherapy, biomarkers related to checkpoints, and immunophenoscore.

Highly expressed lncRNAs are considered to have important biological functions. Tang et al.^38^analyzed the expression levels of five lncRNAs in breast cancer patients, and constructing a model that can predict survival characteristics. We used an algorithm that allows the identification of DEirlncRNA allowing the selection of the most important irlncRNA pairs. Thus, only pairs with increased or decreased levels of expression are considered rather than expression levels of the total lncRNA population. The novel model that we have designed has the advantage of clinical practicality in distinguishing high‐risk or low‐risk clinical cases, compared with a model based on total lncRNA expression. Some lncRNAs included in our model are associated with immune‐related genes and may be involved in the regulation of the tumor immune microenvironment or of immune activation cells. Shen et al.^39^ investigated the characteristics of 10 irlncRNAs in breast cancer, constructed a risk model to assess the high and low risks of patients, and conducted effective verification. Moreover, some of the DEirlncRNAs in our model are known to play an important role in the malignant phenotypes of various cancer types,^40^ whereas others are reported to fulfill this role for the first time during the current study. Liu et al.^41^ has reported previously that lncRNA OSTN‐AS1 was expressed in triple‐negative breast cancer and related to the immune activity in tumors, which may represent that it has become a new type of immune‐related prognostic marker in this type of tumor. Moreover, de Santiagoe revealed that LINC00944 can be regulated and affected by ADAR1 in breast cancer cells, and this lncRNA was strongly related to the immune signal pathway.^42^

Traditional approaches to the construction of models for risk assessment employ the median to distinguish risks. In contrast, we calculated each AUC value to determine maxima and construct the best model by comparing it with other clinical parameters. We also used the AIC value to obtain the best cutoff point for the model fit. Moreover, to improve the accuracy and ensure the effectiveness of risk prediction, we used the improved Lasso penalized model proposed by Sveen et al.^43^ The Lasso penalized model includes factors in the Cox regression process based on the frequency of occurrence rather than on the occurrence itself. Intersection of frequencies suggests the influence of a factor on the model. By means of this improvement, we are able to present a novel re‐evaluation of survival of high‐risk and low‐risk populations. Various results indicated that our modeling algorithm has validity.

It is acknowledged that the infiltration of tumors by immune cells influences anti‐checkpoint blockade. In a clinical trial of IMpassion130, the PFS phase and OS phase of patients with simultaneous high expression of CD8+ TILs and PD‐L1 were significantly improved.^44^ We used seven common acceptable methods to identify and quantify infiltration by immune cells. Our integration analysis results revealed a negative correlation between DEirlncRNA pairs and tumor‐infiltrating immune cells, such as B cells, CD4+ T cells, and CD8+ T cells. In a pooled analysis of 9125 patients from six breast cancer clinical studies, tissue, wax or pathological specimens from 3771 patients were screened for TILs. Among the samples, 44% had a low density of TILs, 36% had moderate density, and 19% had high density. TILs were highly expressed in TNBC and HER2‐positive breast cancer patients (30% and 19%, respectively) but were lower in luminal breast cancer patients (13%).^45^ We speculate that this result may be extrapolated to the majority of patients with luminal breast cancer in the cohort. The model we present indicated that breast cancer patients in the low‐risk group show sensitivity to chemotherapeutic agents, such as doxorubicin, cisplatin, gemcitabine, AZD2281 (Olaparib), and mitomycin C, PD 0332991 (Palbociclib). Although only 20–40% of patients benefit from these new therapies at present, we believe that further research will allow the potential of immunotherapy to exceed that of traditional chemotherapy. Our results demonstrate the expression of ICI‐related biomarkers (PDCD1, LAG3, CTLA4, CD274) among patients was higher in the low‐risk group. Interestingly, in our further immunotherapy analysis, the low‐risk group received higher scores in all four types of immunophenoscore. This may indicate that patients with low‐risk scores are more suitable candidates for immunotherapy. Several clinical studies of neoadjuvant therapy have shown that high expression of PD‐L1,^46^either as mRNA or as protein, is an independent positive predictor of pathological response.^47^ The expression of PD‐L1 is also used by researchers as an indicator of breast cancer survival and prognosis. However, many controversies still remain. In a study on 870 breast cancer patients, Qin et al.^48^ found that the disease‐free survival (DFS), metastasis‐free survival (MFS), and OS of patients with high PD‐L1 expression were all lower when compared with patients with negative expression of PD‐L1. These results seem to indicate that PD‐L1 expression is an indicator of poor prognosis in breast cancer patients.

In spite of our positive findings, we recognize that the current study has some limitations. Although the data set of TCGA has been standardized, the sample size of the breast cancer patients dataset is relatively small. Moreover, we were unable to obtain data sets where lncRNA expression levels, clinicopathological characteristics, and survival outcomes coincided for breast cancer patients. As a result, sample sizes for the survival outcome and clinical characteristics data are not consistent and this may lead to biased results. Furthermore, expression levels differ among samples necessitating verification of the model by an external data set. This may lead to the unreliability of the model. In order to minimize sample errors caused by expression differences, all lncRNA pairs were assigned a value of 0–1 in our matrix. The new modeling algorithm has also been validated by a variety of methods. Although we acknowledge that verification by an external clinical data set would be beneficial, we still believe that our model has utility. We plan to continue our work to expand the sample size and further verify our model.

In summary, the current study presents novel findings regarding the use of irlncRNA without the need to predict precise expression levels in the prognosis of breast cancer patients and to indicate their suitability for anti‐tumor immunotherapy.

## CONFLICT OF INTEREST

The authors declare that they have no conflicts of interest.

## ETHICS STATEMENT

All data of this study were public and required no ethical approval.

## Supporting information

Figure S1

Figure S2

Table S1

Table S2

Table S3

Table S4

Table S5

Table S6

## Data Availability

The data used to support the findings of this study are included in the article.
