# Supplementary figures and images for: Predicting the immune landscape of invasive breast carcinoma based on the novel signature of immune‐related lncRNA
Source: Cancer Med. 2021 Aug 11;10(18):6561–75. doi: 10.1002/cam4.4189 (PMC8446415; doi:10.1002/cam4.4189)

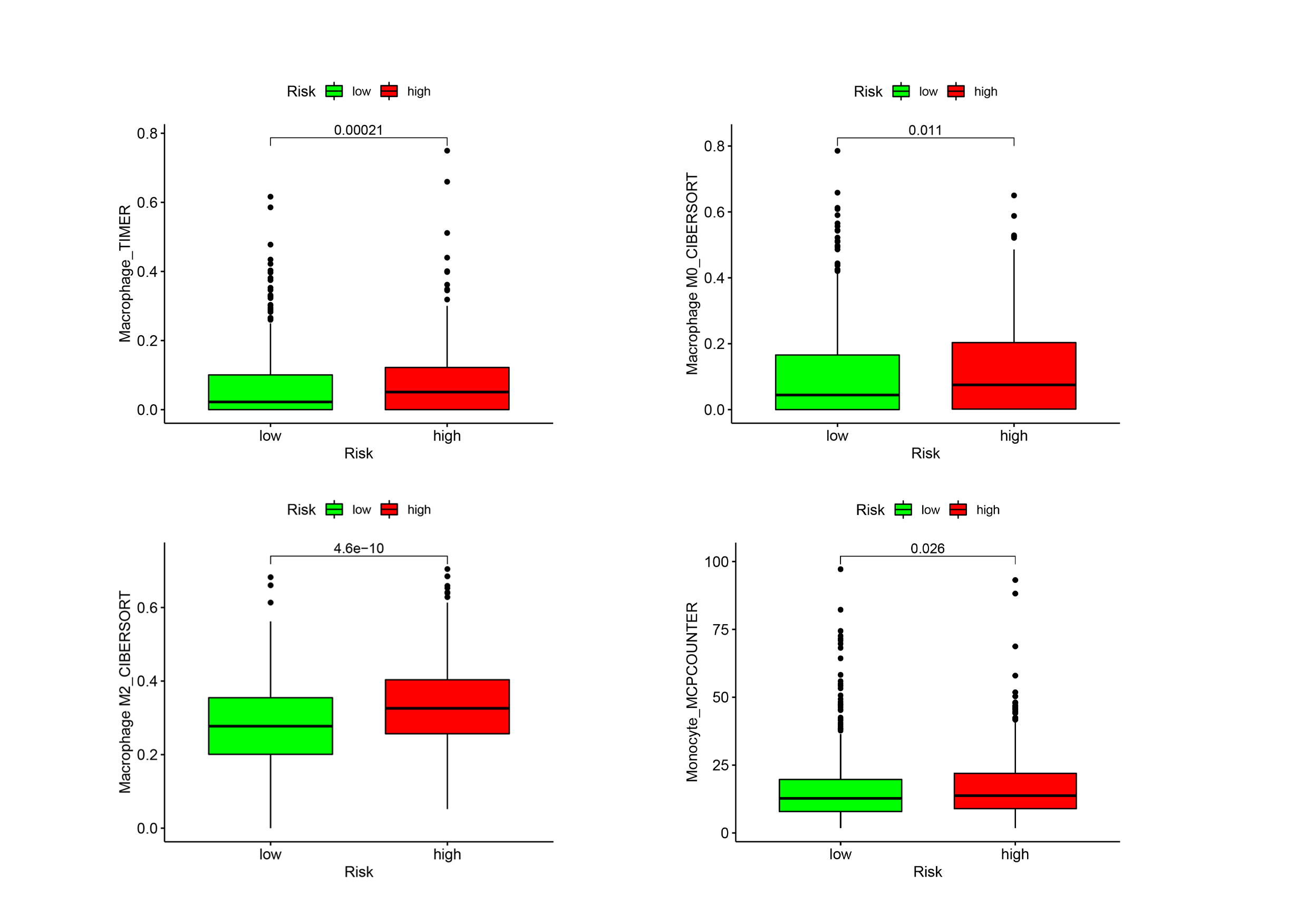

Supplement: Supplementary file 1 — Figure S1 [file CAM4-10-6561-s008.tif]

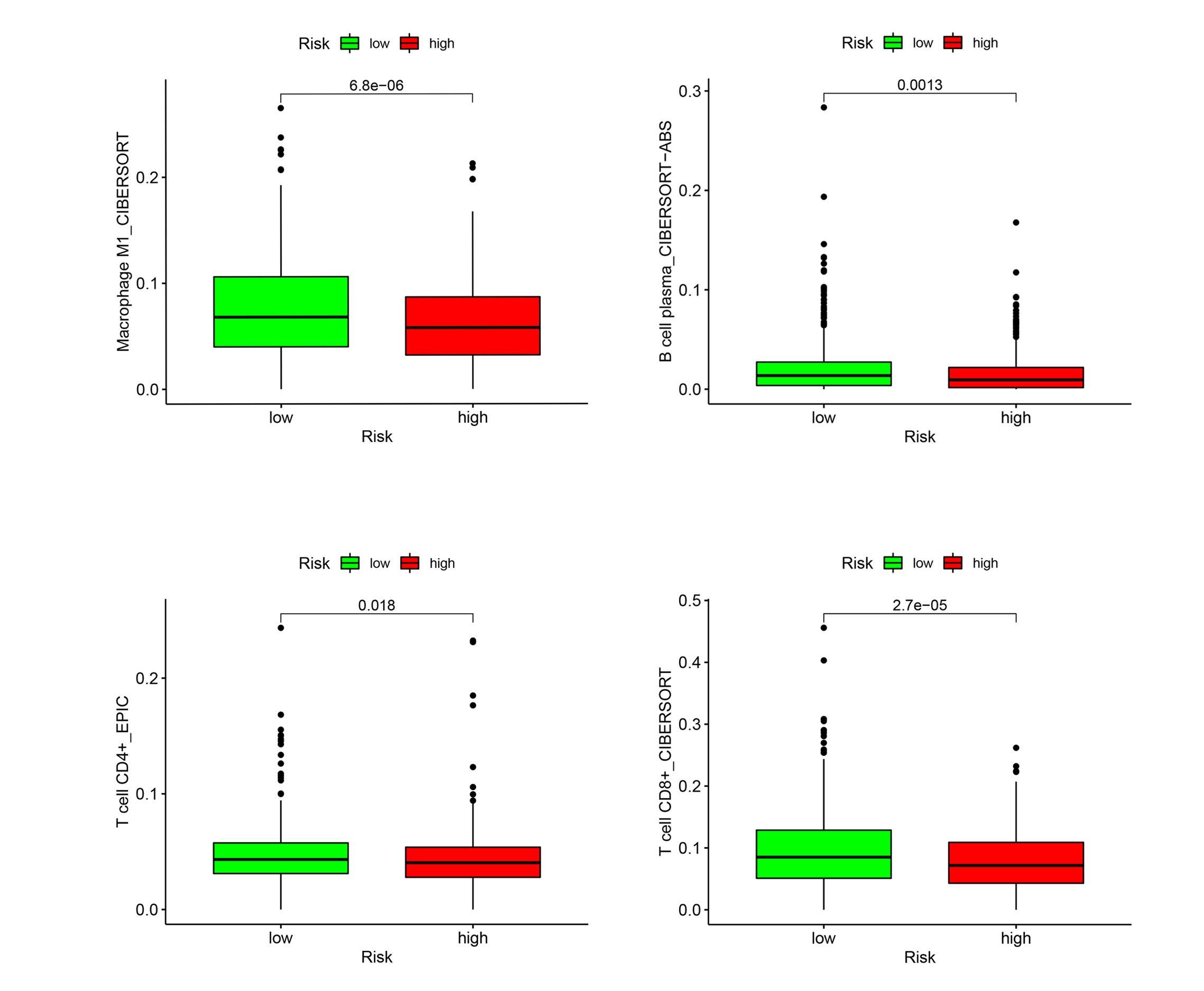

Supplement: Supplementary file 2 — Figure S2 [file CAM4-10-6561-s003.tif]
